# Early Developmental Screening and a Home-Based Caregiver Intervention for Infants and Toddlers With Sickle Cell Disease: Protocol for a Prospective Mixed Methods Study

**DOI:** 10.2196/104084

**Published:** 2026-08-14

**Authors:** Catherine R Hoyt, Hunter G Moore, Ashley J Housten, Ana A Baumann, Allison A King

**Affiliations:** 1 Program of Occupational Therapy, Department of Neurology, Department of Pediatrics, Washington University School of Medicine, 4480 Clayton Ave, St. Louis, MO, 63110, United States, 1 3142861761; 2 Program in Occupational Therapy, Washington University School of Medicine, St. Louis, MO, United States; 3 Program in Occupational Therapy, Division of Public Health Science, Department of Surgery, Washington University School of Medicine, St. Louis, MO, United States; 4 Division of Public Health Science, Department of Surgery, Washington University School of Medicine, St. Louis, MO, United States; 5 Department of Pediatrics, Department of Medicine, Department of Education, Department of Surgery (Prevention and Control), Program in Occupational Therapy, Washington University School of Medicine, St. Louis, MO, United States

**Keywords:** sickle cell disease, child development, early intervention, pediatric, evaluation, development

## Abstract

**Background:**

Sickle cell disease (SCD) is the most common monogenic disorder in humans and occurs predominantly among individuals who identify as Black or African American in the United States. In earlier work, we found that developmental delays were present in more than 50% of children with SCD before the age of 3 years, yet none had been diagnosed or referred to intervention services. Children whose caregivers participated in a home-based caregiver education program demonstrated improved scores on standardized developmental measures. When developmental delays go unidentified, children miss a critical opportunity for intervention during a period of rapid neurological change. Yet few, if any, studies have described the incidence and severity of developmental delays among children with SCD compared to controls.

**Objective:**

The purpose of this study is to determine the incidence and severity of developmental delays in children with SCD under 3 years of age (aim 1), test a 12-month home-based Sickle Cell Collaboration for Child Development (SCCCD) intervention (aim 2), and conduct a mixed methods study to identify contextual determinants to prepare for future scaling of the SCCCD intervention across health care systems (aim 3).

**Methods:**

Consistent with American Academy of Pediatrics guidelines, children with SCD will be evaluated at 9, 18, and 30 months using the Bayley Scales of Infant Development, Fourth Edition, to determine the incidence of developmental delay over the first 3 years of life compared to demographically matched peers (n=100, aim 1). The SCCCD intervention, adapted from a pilot study, combines skilled occupational therapy, the Parents as Teachers curriculum, and SCD-specific caregiver education, delivered over 12 monthly home visits (n=25, aim 2). Interviews with caregivers who participated in and those who declined the intervention will identify contextual determinants (ie, facilitators and barriers) to inform future testing and broader implementation of the SCCCD (aim 3).

**Results:**

This project has been approved by the Institutional Review Board at Washington University School of Medicine (202104034 and 202407080). As of May 31, 2026, a total of 50 participants (26 children with SCD and 24 typically developing children for the comparison cohort) have participated for aim 1. Aim 2 recruitment began in July 2025, and 5 caregiver/child dyads have participated so far. The study is expected to be completed by 2028.

**Conclusions:**

These findings will provide the first prospective characterization of developmental trajectories in children with SCD across the first 3 years of life and establish preliminary evidence for a disease-specific, home-based intervention to improve developmental outcomes in this underserved population. The results will directly inform a future randomized controlled trial of the SCCCD intervention.

## Introduction

Sickle cell disease (SCD) is the most common monogenic disorder in the United States and is associated with severe pain, frequent hospitalizations, stroke, and cognitive decline [1-3]. In the United States, SCD is identified at birth and occurs predominantly among individuals who identify as Black or African American, a racially minoritized group in the United States [4]. Children with SCD are at elevated risk for developmental delays, which can emerge as early as 9 months of age and most often affect communication and cognition [5,6].

The American Society of Hematology (ASH) and the American Academy of Pediatrics both recommend early, frequent developmental screening for children with SCD, with ASH guidelines citing prevalence estimates of developmental delay affecting 30% to 50% of children [6-11]. Despite these recommendations, screening in primary care settings is not standard [12]. Many providers do not feel confident overseeing health care for children with SCD and are therefore less likely to monitor for early, subtle developmental delays [13,14]. Further, children of racially minoritized groups are less likely to be diagnosed with delays even when objective assessment criteria are met [15], and many children with SCD are not referred to intervention services before the age of 3 years. Current practice falls short of both the ASH and American Academy of Pediatrics standards, representing a missed opportunity for timely intervention during a period of rapid developmental change.

Yarboi et al [16] proposed that developmental delays in SCD result from both biological and environmental factors, including the effects of SCD along with household income, caregiver education, and parenting knowledge. Intervention development must consider both physiological and environmental contexts as lower caregiver stress is associated with better cognitive function in children with SCD [16], and greater caregiver knowledge of development can reduce stress, strengthen the caregiving relationship, and improve the home environment [17-19]. Caregivers of children with SCD have expressed a preference for home-based services, citing convenience and accessibility [20]. For this population, well-implemented, higher-dosage, home-based interventions in the first 3 years of life offer the potential to improve cognitive development and reduce the developmental gap between children from lower- and higher-income households [21-24]. Building on this evidence, our laboratory adapted the Parents as Teachers (PAT) curriculum specifically for families of children with SCD. The PAT curriculum is evidence-based and focuses on supporting and engaging caregivers in the home environment to promote optimal early development for children 0 to 3 years of age [21].

In earlier work, we adapted the PAT curriculum and combined it with SCD education and individualized occupational therapy (OT) to monitor and address developmental delays (n=43) [20]. This multicomponent OT + PAT + SCD intervention, the Sickle Cell Collaboration for Child Development (SCCCD), was offered to families until the child aged out of the program at 3 years. Using the Bayley Scales of Infant Development, Third Edition [25], we found that 50% of children over 12 months of age with SCD had a developmental delay, yet none were receiving these therapy services [6]. Caregivers demonstrated greater than 75% adherence with scheduled visits [20], and children whose caregivers participated either maintained or improved their Bayley scores [26]. Caregivers of the youngest children (under 1 year) were the most likely to enroll, and approximately 12 to 13 monthly visits produced the observed developmental gains [20]. These findings indicate that developmental delay in SCD emerges early, is not solely attributable to biological disease burden, is shaped by the caregiving environment, and can be meaningfully modified with approximately 1 year of targeted intervention.

Despite the high prevalence of developmental delay in this population, critical gaps remain. No study has prospectively characterized developmental trajectories across the first 3 years of life in children with SCD using a demographically matched comparison cohort and psychometrically robust assessment methods. Earlier work suggests that delays may worsen as children age [6,7,27], but this trajectory has not been rigorously evaluated. Additionally, no disease-specific intervention has been formally tested for infants and toddlers with SCD, and little is understood about the contextual factors that would support broader implementation of such a program. Because known environmental mediators of development—including socioeconomic status and disease severity—require comparison to well-matched peers, a prospective design with a demographically balanced comparison cohort is essential.

The purpose of this study is to determine the incidence and severity of developmental delays at 9, 18, and 30 months of age in children with SCD compared to demographically matched peers (aim 1); evaluate the effect of a 12-month, home-based SCCCD intervention on developmental outcomes in children with SCD (aim 2); and identify contextual determinants that facilitate or impede caregiver participation in developmental screening and home-based intervention, in preparation for future broader implementation (aim 3).

## Methods

### Study Design and Setting

This study is funded by the National Heart, Lung, and Blood Institute under a K23 award designated Independent Clinical Trials Not Allowed (K23HL161328), reflecting the determination by the National Institutes of Health that the proposed research does not constitute an independent clinical trial. Aim 1 is an observational cohort study, and aim 2 is a feasibility pilot intended to inform a future randomized controlled trial. Accordingly, this study was not registered on ClinicalTrials.gov. This study includes 3 aims with different designs, and we selected a reporting guideline for each. We follow the STROBE (Strengthening the Reporting of Observational Studies in Epidemiology) guideline for the aim 1 cohort [28], the TIDieR (Template for Intervention Description and Replication) for the aim 2 SCCCD intervention [29], and the COREQ (Consolidated Criteria for Reporting Qualitative Research) for the aim 3 interviews [30]. We report the protocol following the SPIRIT (Standard Protocol Items: Recommendations for Interventional Trials) 2025 statement [31]. Completed TIDieR and SPIRIT 2025 checklists are provided as Checklists 1 and 2, respectively.

Participants are recruited through Washington University School of Medicine and the Sickle Cell Clinic at St. Louis Children’s Hospital (SLCH), a regional hub for pediatric SCD care that follows over 400 children and receives approximately 25 newborns with SCD annually. For aim 1, we will recruit 100 children, 50 with SCD and 50 demographically matched peers, who will complete developmental evaluations at 9, 18, and 30 months with concurrent caregiver surveys. For aim 2, an additional 25 children with SCD will be recruited to participate in a 12-month home-visiting intervention. Caregivers of children enrolled in aim 2 may also be invited to participate in a qualitative interview as part of aim 3.

### Participants

#### Aim 1: Incidence Cohort (n=100)

Children with SCD (n=50) under 9 months of age with all SCD genotypes and their caregivers will be recruited from the Sickle Cell Clinic at SLCH. Children without SCD (n=50) will be recruited as a comparison cohort and matched at the group level to the SCD cohort on the distributions of age, sex, race, socioeconomic status, and Area Deprivation Index [32], rather than paired individually. Group-level matching will be finalized after enrollment. Typically developing children will be recruited through SLCH general medicine, community early childhood centers, local pediatricians, public libraries, local early intervention providers, and faith-based organizations, using methods previously successful in this lab [33,34]. Children will be excluded if they have a diagnosis associated with developmental delay that is not attributable to SCD, or if the family is not English-speaking.

Caregivers of children with SCD will be approached by phone or following a regularly scheduled clinic visit. Interested caregivers will review the informed consent document with a member of the research team by phone, Health Insurance Portability and Accountability Act (HIPAA)–secure video call, or in person.

#### Aim 2: Intervention Cohort (n=25)

An additional 25 infants with SCD under 9 months of age will be recruited for the SCCCD intervention following the same inclusion and exclusion criteria as aim 1. Following the 9-month baseline evaluation, consenting families will participate in the 12-month SCCCD program.

#### Aim 3: Qualitative Interview Sample (n=35)

Up to 35 caregivers of children with SCD will be invited to participate in a semistructured interview. Participants will be drawn from three groups: caregivers who completed 6 or more SCCCD intervention visits, caregivers who completed fewer than 6 visits, and caregivers who declined the intervention. With the consent of the primary caregiver, additional caregivers may also be invited to participate. Recruitment will continue until thematic saturation is reached [35].

### Procedures

Developmental evaluations will be conducted at 9, 18, and 30 months of age (±1 month) for children enrolled in both aim 1 and aim 2. Evaluations will take place in a dedicated child-friendly testing room on the Washington University School of Medicine campus, which has a padded floor, child-safe furniture, and space for caregivers to sit comfortably. Five accessible parking spots are reserved directly in front of the testing room, and the space is located within a 5-minute walk of public transportation. Visits will last approximately 60 to 90 minutes. Snacks will be provided to support child engagement during the evaluation, and caregivers will be compensated for their time and travel at each visit. Following institutional review board (IRB) amendment, evaluations may also be conducted in the family’s home for participants residing within the Greater St. Louis Metro area, in response to caregiver feedback regarding barriers to on-site attendance. Any changes are communicated to participants and shared with our community advisory board.

Demographic information and caregiver surveys will be collected using REDCap, a HIPAA-secure web-based data capture system, administered on a research tablet [36,37]. Survey data may also be completed by caregivers remotely via a secure web-link before or after the visit to reduce in-person visit burden. Assessors will be trained to maintain adherence to the evaluation protocol prior to data collection. Following each evaluation, the assessor will prepare a written summary report of the child’s developmental scores, review the results with the caregiver, and share the report with the child’s pediatrician and hematologist at the family’s request. If a developmental delay is identified (>1 SD below the mean), the research team will discuss the results with the caregiver and offer a referral to Early Intervention services, the federally funded, state-administered early intervention program available to eligible children under 3 years of age at no cost to families [38,39].

Retention strategies include SMS text message reminders prior to each scheduled visit; flexible scheduling, including early morning, evening, and weekend appointments; and reimbursement for parking or public transportation. Text message reminders have been effective in reducing visit cancelations in our prior work [20].

For aim 2, a pediatric occupational therapist with training in the PAT curriculum will conduct 12 monthly home visits with each enrolled family following the 9-month baseline evaluation. Visit scheduling will allow for up to 15 to 18 months of delivery to accommodate missed visits and ensure all families receive equivalent intervention dosage. Visits will be documented using daily notes that capture completed activities, caregiver engagement, visit duration, and any SCD-related topics reviewed.

For aim 3, semistructured interviews will be conducted with up to 35 caregivers of children enrolled in aim 2. Interviews will be conducted by the method of the participant’s choosing (eg, phone, HIPAA-secure video call, or in person) and are expected to last approximately 30 to 45 minutes. Interviews will be audio-recorded and transcribed verbatim. Two research team members will review all transcripts for accuracy prior to analysis. Caregivers will be compensated for their time.

All data will be stored with 2 levels of password protection. Paper consent documents will be maintained in a locked cabinet in a locked office. Data will be accessible only to the research team.

### Measures

All outcome measures are summarized in Table 1. Demographic information will be collected from all participants at enrollment, including caregiver age, education, health literacy, marital status, household income, racial or ethnic identity, and number of children in the household. Clinical information collected for children with SCD will include SCD genotype, insurance status, hospitalizations and treatments, disease-modifying medication use, and neuroimaging results if available.

**Table 1.** Summary of outcome measures.

Measure	Purpose	Type	Time point (months)	Aim
Bayley-4^a^	Child development	Clinician administered	9, 18, and 30	1 and 2
ASQ-3^b^	Child development	Caregiver report	9, 18, and 30	1 and 2
ITACS^c^	Child participation in family routines	Caregiver report	9, 18, and 30	1 and 2
BRIEF-P^d^	Executive functioning	Caregiver report	30 only	1 and 2
HOME^e^ Inventory	Home environment	Clinician observation	9, 18, and 30	2
PROMIS^f^ Depression (8 items)	Caregiver mental health	Caregiver report	9, 18, and 30	1 and 2
Semistructured interview	Implementation determinants	Interview	Ongoing	3

a Bayley-4: Bayley Scales of Infant Development, Fourth Edition.

b ASQ-3: Ages and Stages Questionnaires, Third Edition.

c ITACS: Infant Toddler Activity Card Sort.

d BRIEF-P: Behavior Rating Inventory of Executive Function-Preschool.

e HOME: Infant Toddler Home Observation for Measurement of the Environment.

f PROMIS: Patient-Reported Outcomes Measurement Information System.

The Bayley Scales of Infant Development, Fourth Edition (Bayley-4) is the primary outcome measure and has been the gold standard for evaluating and identifying developmental delay in children for over 50 years [25]. The Bayley-4 is completed in 45 to 60 minutes and evaluates 5 developmental domains: cognition, fine motor, gross motor, receptive language, and expressive language. Raw scores are converted to scaled scores, where a score of 10 indicates performance at the 50th percentile. A score greater than 1 SD below the mean will be classified as a developmental delay.

The Ages and Stages Questionnaire, Third Edition (ASQ-3) is a caregiver report screener used widely in primary care and research settings that assesses development across the domains of communication, gross motor, fine motor, problem solving, and personal-social skills in approximately 10 minutes [10,40]. While the ASQ-3 is time- and cost-efficient, it is insufficiently detailed to identify early indicators of mild-to-moderate developmental delays when used alone [5,10,18,20,41,42].

The Survey of Well-Being of Young Children (SWYC) is a brief, validated caregiver report screener that captures developmental and behavioral milestones as well as family context factors including caregiver depression and social determinants of health [43,44]. The SWYC was added to the protocol following community partner and community advisory board input to capture additional environmental and contextual factors and to allow comparison with other commonly used screening measures.

The Infant Toddler Activity Card Sort (ITACS) is a picture-based caregiver report tool developed and validated by members of this team to identify concerns related to child participation in family activities and routines [33,34,45]. Caregivers are presented with photographs depicting common infant and toddler activities and asked to identify those that present a challenge for their child.

The Behavior Rating Inventory of Executive Function-Preschool (BRIEF-P) is a widely used caregiver report measure of executive functioning for children as young as 2 years of age, designed to identify how a child functions within the context of their individual environments [46]. The BRIEF-P will be administered at the 30-month time point only.

The Infant Toddler Home Observation for Measurement of the Environment Inventory [9] is a 42-item checklist completed by a research team member that evaluates the quality and quantity of stimulation and support available to the child in the home environment [47]. The HOME (Infant Toddler Home Observation for Measurement of the Environment) will be administered at all 3 time points for children enrolled in aim 2.

The Patient-Reported Outcomes Measurement Information System (PROMIS) Depression Short Form (8 items) will be completed by caregivers at each time point to assess caregiver mental health, given the established association between caregiver depression and child developmental outcomes [16,48,49]. Caregivers with concerning scores on the PROMIS Depression screener, based on prespecified cutoffs, will be referred to behavioral health services.

### Intervention (Aim 2)

The SCCCD intervention is a multicomponent, home-based program delivered by a pediatric occupational therapist trained in both the PAT curriculum and SCD care. Following our previously successful protocol [20], the intervention integrates 3 components during monthly home visits: OT to monitor and address developmental delays, the PAT curriculum to support caregivers in promoting developmental milestones and positive parenting, and SCD-specific education covering clinical recommendations such as medication adherence and protective behaviors (eg, hydration). Delivery by a single provider across all 3 components is intentional, as this approach establishes a high level of rapport between the family and the interventionist over the course of the program.

The intervention is adapted from our pilot study to be feasible within this population while maintaining sufficient dosage to observe change [20]. Families of infants under 9 months of age will be enrolled, and 12 monthly home visits will be offered over the course of 1 year [50-52]. In practice, we anticipate that some families will miss visits due to health-related concerns associated with SCD; therefore, the program may extend up to 15 to 18 months to ensure all children receive equivalent intervention dosage. In our prior work, maintaining frequent contact with families through their preferred communication method (eg, SMS) increased visit completion to 80% [20]. The PAT curriculum provides a developmentally appropriate activity and caregiver handouts for each visit. OT during visits will target specific developmental delays identified through ongoing evaluation, and SCD education will be reviewed and updated at each contact.

OT is uniquely suited to deliver this intervention given the combined expertise in mental health, rehabilitation, and child development. The proposed SCCCD intervention package, which combined OT, PAT, and SCD education, reflects a theoretically grounded, practically feasible approach to improving developmental outcomes in a population with both biological and environmental risk factors. The intervention will be discontinued if the family requests to stop services.

### Fidelity Monitoring

Interventionists will be trained to maintain adherence to the SCCCD protocol prior to delivering visits. Using the Comprehensive Intervention Fidelity Guide, the intervention design, training, and delivery will be systematically evaluated [53]. Fidelity will be documented through daily visit notes that capture completed activities, caregiver engagement, visit duration, handouts provided, SCD topics reviewed, and any additional caregiver training or referrals made [54,55]. Visits scheduled and completed will be tracked throughout the intervention period.

### Implementation Science Framework (Aim 3)

To identify determinants that influence caregiver participation in developmental screening and the SCCCD intervention, we will use a mixed methods (QUAL → quan) approach guided by the Consolidated Framework for Implementation Research 2.0 (CFIR) [56-58]. The CFIR is one of the most widely used frameworks for understanding contextual determinants in implementation research and has been recently updated with greater emphasis on including recipients of the intervention as key informants [56], making it well suited for a caregiver-centered inquiry.

Semistructured interviews will be conducted with up to 35 caregivers of children enrolled in aim 2, organized around 3 CFIR construct domains: outer setting (eg, health care system and community context), intervention characteristics (eg, acceptability and perceived fit of the SCCCD), and inner setting (eg, family and social support). We are confident that thematic saturation will be reached [35,59]. Qualitative themes from interviews will be integrated with quantitative participation data from aim 2—including visit completion rates, home visit duration, and family engagement—to provide a comprehensive characterization of the contextual determinants that facilitate or impede caregiver participation.

Interviews will be audio-recorded and transcribed verbatim. Two research team members will use an iterative inductive and deductive thematic analysis approach to review all transcripts and generate themes for a preliminary codebook [60]. The preliminary codes will be applied to 4 interviews using NVivo software (version 14; Lumivero) [61]; any deviations will be discussed, and the revised codebook will be applied to all remaining interviews [62]. Codes will be analyzed for themes, and participation data from aim 2 will be summarized [63,64].

### Data Analysis

#### Aim 1

Evaluations will be scored according to their respective manuals. Mixed effects regression will be used to analyze developmental outcome data, with time, group, and time-by-group interaction entered as fixed effects. This approach accounts for within-subject and within-group variability across the 3 longitudinal time points. Scores from the Bayley-4 will be compared to the ASQ-3. Covariates will include race, age, sex, caregiver education, socioeconomic status, disease-modifying medication use, and therapeutic intervention received. The number of evaluations completed and any therapeutic or behavioral interventions received (eg, physical therapy, OT, speech-language therapy) will be documented. Children who receive therapy services during the study period will be analyzed separately.

#### Aim 2

Standardized scores will be calculated for each assessment according to the respective manuals. Scores greater than 1 SD below the mean will be classified as a developmental delay. Repeated measures ANOVA will be used to compare group mean scores at 9, 18, and 30 months. Logistic regression will compare the number of children classified with a delay at 30 months to the aim 1 cohort. The number of caregivers approached and enrolled will be documented, and visits scheduled and completed, along with topics from visit notes, will be summarized. Additional analyses will examine possible changes in the home environment and caregiver mental health before and after the intervention. When the outcome is binary, a mixed effects logistic regression will be used, with time, group, and time-by-group interaction entered as fixed effects and variation of subject intercept as a random effect. Time will also be tested as a random effect in a more elaborate model.

#### Aim 3

Two research team members will use an iterative inductive and deductive thematic analysis approach to review all transcripts and generate themes for a preliminary codebook [60]. The preliminary codes will be applied to 4 interviews using NVivo software; any deviations will be discussed, and the revised codebook will be applied to all remaining interviews [62]. Codes will be analyzed for themes, and participation data from aim 2 will be summarized and integrated with qualitative findings [63,64].

The study protocol and deidentified data will be made available from the corresponding author on request and consistent with the NIH Data Management and Sharing Plan.

### Missing Data and Attrition

Participants who do not complete all evaluation time points will be analyzed using available data. Attrition will be documented across all aims, including the number of participants who withdraw, are lost to follow-up, or miss individual evaluation visits, and patterns of missingness will be reported.

### Ethical Considerations

This study has been approved by the Human Research Protection Office at Washington University School of Medicine (IRB 202104034 and 202407080), approved April 2021 and July 2024, respectively. This study poses minimal risks to children and families. Study personnel will complete mandated reporter training, and caregivers will be referred to supportive services if any psychological, environmental, or developmental concerns arise. All study procedures will be conducted in accordance with the ethical standards of the IRB.

Informed consent will be obtained from the parent or legal guardian for themselves and on behalf of the child before any study procedures. The consent process will take place in a private setting and will be conducted in person, by phone, or via HIPAA-secure video call. Caregivers will be given time to ask questions and will receive a copy of the signed consent document. Since all child participants are under 3 years of age, child assent will not be obtained. Caregivers may withdraw from the study at any time without consequence.

All data will be stored with 2 levels of password protection. Paper consent documents will be maintained in a locked cabinet in a locked office on the Washington University School of Medicine campus. Digital data will be collected via REDCap and will be accessible only to members of the research team. Any identifying information will be coded so that it can only be linked to individual participants by the research team.

The risks associated with this study are no greater than those encountered during a routine clinical or therapy visit. Caregivers may experience mild stress or anxiety when discussing their child’s development. To mitigate this, all research team members will be trained in strategies for communicating sensitively about developmental concerns with caregivers. All research staff interacting with participants will also complete mandatory reporter training for child abuse and neglect. Caregivers will have the option to complete surveys verbally if preferred. Caregivers with concerning scores on the PROMIS Depression screener will be referred to behavioral health services.

## Results

A total of 128 caregivers have been approached, and 72 have consented. Of those, 13 participants withdrew because of time constraints, interest, or loss to follow-up. As of May 31, 2026, a total of 26 unique participants with SCD have participated in aim 1, and 24 unique participants participated in the comparison cohort. For aim 2 with the pilot intervention, 13 caregivers have been approached and 7 have consented, and 5 caregiver or child dyads have been enrolled and have participated. Two caregivers withdrew due to moving out of state or living outside of the intervention radius (>60 miles). Characteristics of participants to date are available in Tables 2 and 3. Enrollment data can be seen in Table 4.

**Table 2.** Caregiver demographics.

Demographics	Sickle cell cohort (aim 1; n=26)	Typically developing cohort (aim 1; n=24)	Sickle cell cohort (aim 2; n=5)
Parental role			
Mother, n	23	22	5
Father, n	3	2	0
Age (y), mean (SD)	30.9 (7.4)	33.4 (5.0)	28.8 (7.9)
Age group (y), n			
20‐29	17	6	2
30‐39	5	15	3
40‐49	4	3	0

**Table 3.** Child demographics.

Demographics	Sickle cell cohort (aim 1), n	Typically developing cohort^a^ (aim 1), n	Sickle cell cohort (aim 2), n
Gender			
Male	13	13	3
Female	13	11	2
Race or ethnicity			
White	0	15	0
Black or African American	25	7	5
Asian	0	1	0
More than one race	1	1	0
Hispanic	0	2	0
Area of Deprivation Index state values			
1‐4 (least deprived)	6	14	1
5‐7	6	5	2
8‐10 (most deprived)	14	4	2

a One participant is excluded in Area Deprivation Index due to being suppressed in the calculation. Values reflect interim enrollment.

**Table 4.** Current study participant enrollment (May 31, 2026).

Enrollment	Sickle cell disease (aim 1)	Typically developing (aim 1)	Sickle cell disease (aim 2)
9 months	12	10	5
18 months	22	15	0
30 months	18	16	0
Total unique participants	26	24	5
Total approach	84	44	13
Total consent	35	37	7

Participants in aim 1 for the SCD cohort all identify as Black or African American; 7 participants in the comparison cohort identify as Black or African American, 15 identify as White, 1 identifies as Asian, and 1 identifies as more than one race.

Aim 1 enrollment is ongoing; 50 participants are needed to fulfill the recruitment goal of aim 1 (n=100), 24 more in the SCD cohort, and 26 more in the comparison cohort. Enrollment in aim 2 began in July 2025, and 20 more participants will be recruited to this pilot intervention. As of May 31, 2026, one participant has initiated and completed 5 home visits.

## Discussion

### Anticipated Findings

This study will provide the first prospective characterization of developmental trajectories in children with SCD across the first 3 years of life, using a psychometrically robust assessment battery and a demographically matched comparison cohort. Despite evidence that developmental delays affect at least 50% of young children with SCD [6,26], the trajectory of these delays from infancy has not been rigorously evaluated, and no disease-specific intervention has been formally tested in this age group. The SCCCD intervention combines OT, the PAT curriculum, and SCD-specific caregiver education and is adapted from a pilot protocol with demonstrated feasibility and preliminary effectiveness in this population [20,26]. The findings from this study will establish preliminary evidence for the SCCCD as a structured, home-based approach to improving developmental outcomes in children with SCD and will generate the implementation science evidence needed to support broader scale-up.

### Protocol Adaptations

Two protocol adaptations were made following IRB approval and are documented here in the interest of transparency. First, in response to caregiver feedback regarding barriers to on-site attendance, evaluations may now be conducted in the family’s home for participants residing within the Greater St. Louis Metro area. This adaptation reflects the access barriers that have historically limited participation of Black and African American families in research and clinical programs and is consistent with the home-based delivery model of the SCCCD intervention. Second, the SWYC [43] was added to the assessment battery following input from community advisory board members, who recommended its inclusion to capture additional environmental and contextual factors and to allow comparison with other commonly used developmental screening measures used in community settings.

### Limitations

Several limitations should be acknowledged. Recruitment is concentrated at a single site, which may limit the generalizability of the findings to SCD programs in other regions. The English-language-only inclusion criterion, necessary given the validation constraints of the assessment measures, excludes non-English-speaking families and limits the diversity of the sample. Aim 2 does not include a randomized comparison condition; developmental outcomes for children who receive the SCCCD intervention will be compared to the aim 1 observational cohort, which introduces the potential for selection bias. Some outcome measures rely on caregiver report, which may be subject to response bias. Finally, participants who do not complete all evaluation time points will be analyzed using available data, and attrition will be documented and reported.

### Conclusions

Early identification of developmental delays in children with SCD remains inconsistent, and the window for intervention in the first 3 years of life is frequently missed [12,15]. This study directly addresses that gap by characterizing developmental trajectories from infancy, testing a home-based intervention designed for this population, and identifying the contextual factors that will inform future implementation. The results will be shared through community partners, peer-reviewed publications, and scientific meetings. The study design reflects a deliberate commitment to reducing structural barriers to participation for families, including home-based delivery, flexible scheduling, transportation support, and ongoing community advisory board engagement. The results will provide the foundation for a future randomized controlled trial of the SCCCD intervention and inform clinical recommendations for developmental screening and early intervention in SCD.

## Supplementary material

10.2196/104084 Checklist 1 TIDieR checklist.

10.2196/104084 Checklist 2 SPIRIT checklist

10.2196/104084 Peer Review Report 1 Peer review report from the National Heart, Lung, and Blood Institute (NHLBI) of the National Institutes of Health (NIH) (K23HL161328).

## Abbreviations

ASH: American Society of Hematology
ASQ-3: Ages and Stages Questionnaire, Third Edition
Bayley-4: Bayley Scales of Infant Development, Fourth Edition
BRIEF-P: Behavior Rating Inventory of Executive Function-Preschool
CFIR: Consolidated Framework for Implementation Research
COREQ: Consolidated Criteria for Reporting Qualitative Research
HIPAA: Health Insurance Portability and Accountability Act
HOME: Infant Toddler Home Observation for Measurement of the Environment
IRB: institutional review board
ITACS: Infant Toddler Activity Card Sort
OT: occupational therapy
PAT: Parents as Teachers
PROMIS: Patient-Reported Outcomes Measurement Information System
SCCCD: Sickle Cell Collaboration for Child Development
SCD: sickle cell disease
SLCH: St. Louis Children's Hospital
SPIRIT: Standard Protocol Items: Recommendations for Interventional Trials
STROBE: Strengthening the Reporting of Observational Studies in Epidemiology
SWYC: Survey of Well-Being of Young Children
TIDieR: Template for Intervention Description and Replication
