# Supplementary material for: Early Developmental Screening and a Home-Based Caregiver Intervention for Infants and Toddlers With Sickle Cell Disease: Protocol for a Prospective Mixed Methods Study
Source: JMIR Res Protoc. 2026 Aug 14;15:e104084. doi: 10.2196/104084 (PMC13475936; doi:10.2196/104084)
Supplement: Supplementary file 1 [file resprot-v15-e104084-s001.pdf]

TIDieR

Sickle Cell Collaboration for Child Development (SCCCD)

Why: Developmental delay in this population reflects both biological disease burden and modifiable environmental factors, including caregiver knowledge, caregiver stress, and the home environment. The SCCCD targets these factors through three components delivered together: occupational therapy to monitor and address developmental delays, the Parents as Teachers (PAT) curriculum to support caregivers in promoting milestones and positive parenting, and SCD-specific education to reinforce disease self-management. Delivery by a single provider across all three components is intentional and builds sustained rapport between the family and the interventionist over the course of the program.

What (material): Each visit uses the Parents as Teachers (PAT) curriculum, which supplies a developmentally appropriate activity and accompanying caregiver handouts, along with SCD-specific educational content covering clinical recommendations such as medication adherence and protective behaviors. The PAT curriculum requires training to access and is available to all affiliates of the program. The SCD specific education materials were co-developed with community partners, SCD care providers, the research team and Health Literacy Media and cover the following topics: what is sickle cell disease, common complications, preparing for and seeking emergency care, SCD related pain, communicating with your child's healthcare team, nutrition and sleep, navigating childcare for your child with SCD, home visits to support child development, how are you doing/reflection, and next steps after home visits.

What (procedures): The interventionist conducts monthly home visits that integrate the three components. Occupational therapy during each visit targets specific developmental delays identified through ongoing evaluation; the PAT activity for that visit is delivered with its caregiver handouts; and SCD education is reviewed and updated at each contact.

Who provided: A single pediatric occupational therapist trained in both the PAT curriculum and SCD care delivers all visits. The interventionist has prior experience evaluating children with SCD and over 20 years of experience providing early intervention in the home environment.

How (mode of delivery; individual or group): Face-to-face, delivered individually to each family.

**Where:**	In the family's home.
**When and how much:**	Twelve monthly home visits are offered over approximately one year following the 9-month baseline evaluation. Because families may miss visits for SCD-related health reasons, delivery may extend to 15–18 months so that all children receive equivalent intervention dosage.
**Tailoring:**	The occupational therapy component is individualized: at each visit, the therapist selects a visit activity from the PAT curriculum that aligns with the child's age and family priorities.
**How well (planned):**	Fidelity will be assessed using the Comprehensive Intervention Fidelity Guide, evaluating intervention design, provider training, and delivery. Fidelity is documented through daily visit notes capturing completed activities, caregiver engagement, visit duration, handouts provided, SCD topics reviewed, and any additional caregiver training or referrals. Visits scheduled and completed are tracked throughout the intervention period.
