# Supplementary material for: Early Developmental Screening and a Home-Based Caregiver Intervention for Infants and Toddlers With Sickle Cell Disease: Protocol for a Prospective Mixed Methods Study
Source: JMIR Res Protoc. 2026 Aug 14;15:e104084. doi: 10.2196/104084 (PMC13475936; doi:10.2196/104084)
Supplement: Supplementary file 3 [file resprot-v15-e104084-s003.pdf]

SUMMARY STATEMENT
(Privileged Communication)

PROGRAM CONTACT:
Dr BENYAM Hailu
301-402-1366
benyam.hailu@nih.gov

Release Date: **08/22/2022**
Revised Date:

Application Number: **1 K23 HL161328-01A1**
Formerly: **1K23HL161328-01**

Principal Investigator

HOYT, CATHERINE ROSE

Applicant Organization: WASHINGTON UNIVERSITY

Review Group: **MPOR (OA)**
NHLBI Mentored Patient-Oriented Research Study Section

Meeting Date: **06/30/2022**
Council: **OCT 2022**
Requested Start: **09/01/2022**
RFA/PA: **PA20-205**
PCC: **BBCCHN5**
Dual IC(s): **HD**

Project Title: **Early Identification Of Developmental Delay Among Infants And Toddlers With Sickle Cell Disease**
SRG Action: **Impact Score:25**
Next Steps: **Visit https://grants.nih.gov/grants/next_steps.htm**
Human Subjects: **30-Human subjects involved - Certified, no SRG concerns**
Animal Subjects: **10-No live vertebrate animals involved for competing appl.**
Gender: **1A-Both genders, scientifically acceptable**
Minority: **2A-Only minorities, scientifically acceptable**
Age: **1A-Children, Adults, Older Adults, scientifically acceptable**

Project Year	Direct Costs Requested	Estimated Total Cost
1	100,030	107,901
2	121,236	130,776
3	139,003	149,941
4	139,183	150,135
5	139,058	150,000
TOTAL	638,510	688,754

ADMINISTRATIVE BUDGET NOTE: The budget shown is the requested budget and has not been adjusted to reflect any recommendations made by reviewers. If an award is planned, the costs will be calculated by Institute grants management staff based on the recommendations outlined below in the COMMITTEE BUDGET RECOMMENDATIONS section.

1 K23 HL161328-01A1 HOYT, CATHERINE

RESUME AND SUMMARY OF DISCUSSION:
This is a resubmission of a K23 application from Dr. Catherine Hoyt, an occupational therapist and rehabilitation scientist (PhD), in which she lays out a plan for becoming an independent scientist with expertise on interventions for developmental deficits among infants and toddlers with sickle cell disease. Reviewers were impressed with the Candidate due to her commitment to the research topic, supportive letters of reference and support of her mentors and institution, and with the unique skills and perspective that she brings to this important and understudied area. Reviewers thought that she was very responsive to the past critiques and the application has been significantly improved by including improved assessment of social determinants of health, PROMIS and measures of executive function (BRIEF-P). However, some weaknesses worth noting remain. Regarding the Research Plan, concerns were raised about recruitment and retention; how the exclusion criteria of "fragile health" will be defined and how it will affect recruitment and retention; some dependency of aims; and the focus on primary care providers and not including hematologist for the intervention. Overall, reviewers were highly confident in the Candidate's potential for becoming an independent researcher and that despite some remaining weaknesses, with the guidance of her mentors, she will be successful in doing so.

DESCRIPTION (provided by applicant):
This K23 application proposes a research and career development plan for Catherine Hoyt, PhD, OTD to establish herself as an independent rehabilitation scientist focused on the early identification and intervention for developmental deficits among infants and toddlers with sickle cell disease (SCD). SCD is the most common monogenic disorder in humans and is primarily inherited by who identify as Black or of African descent. Complications associated with SCD (e.g., infection, pain, stroke) are common in the first years of life. In our earlier work, we found that developmental deficits were present in > 50% of children with SCD before the age of 3 but are none had been diagnosed or referred to intervention services. Further, children whose caregivers participated in a home-based caregiver education program demonstrated improved test scores on standardized measures. Thus, when developmental deficits are overlooked, children miss a critical opportunity for intervention that could improve their developmental trajectory. The American Society of Hematology (ASH) recommends frequent developmental screening starting in the first years of life for all children with SCD. Yet few, if any, studies have described the incidence and severity of developmental deficit among children with SCD compared to controls. Consistent with the American Academy of Pediatrics (AAP) guidelines, this research will evaluate children with SCD at 9, 18, and 30 months using the best available developmental measure, the Bayley Scales of Infant Development-4 (Bayley), to determine the incidence of developmental deficit over the first 3 years of life compared to demographically match peers (n = 100, Aim 1). If developmental deficits are identified, scores will be shared with the child's healthcare team so they can be addressed. Based on theory and evidence, the proposed study will also test a multi-component Sickle Cell Collaboration for Child Development (SCCCD) intervention. The SCCCD combines skilled therapeutic intervention to address developmental deficits, the Parents as Teachers® curriculum and specific SCD education. Our innovative SCCCD intervention is adapted from a pilot study and will provide 12 home visits to caregivers and children with SCD over the course of 1 year (n = 25, Aim 2). Interviews with caregivers who participated, as well as those who declined, will identify contextual determinants (i.e., facilitator and barriers) to prepare for future testing and scaling up of the SCCCD intervention (Aim 3). The results from this K23 award will provide data to understand the onset of developmental deficit in this understudied population and identify the next steps to conduct a randomized control trial to test our SCCCD intervention in an R01 level grant submission. These mentored research aims, combined with a career development plan for advanced training in implementation science (Goal A), mixed methods (Goal B), prospective trial design (Goal C) and professional development (Goals D, E) will enable Dr. Hoyt to launch a career as an independent scientist.

PUBLIC HEALTH RELEVANCE:

Sickle cell disease affects 100,000 people and 2,000 newborns each year; 50% of these children have a developmental deficit (>2 SD) before the age of 3. Early identification of developmental deficit supports timely intervention, but children with sickle cell disease are grossly underdiagnosed and undertreated. The goal of the proposed study is to determine the incidence and severity of developmental deficit at 9, 18 and 30 months of age among children with sickle cell disease and test a 12-month, home-based caregiver intervention with this disproportionately affected population.

CRITIQUE 1:

Candidate: 2
Career Development Plan/Career Goals /Plan to Provide Mentoring: 2
Research Plan: 6
Mentor(s), Co-Mentor(s), Consultant(s), Collaborator(s): 2
Environment Commitment to the Candidate: 2

Overall Impact:
This is a resubmission of a K23 from an Instructor in the Program for Occupational Therapy in the School of Medicine at Washington University. The candidate has been somewhat responsive to the previous critiques. The candidate is clearly committed to a career in patient oriented research to better understand the early neurodevelopmental trajectories of infants and children with sickle cell disease, so that interventions can be designed and implemented to improve their outcomes. The candidate has identified gaps in knowledge and has developed a career development plan that includes 5 training goals that address these gaps. The research plan has 3 specific aims; 1) to determine the neurodevelopmental trajectory of children with sickle cell disease with assessments at 9, 18, and 30 months of age and compare them to a demographically matched cohort; 2) evaluate the effects of a multi-disciplinary intervention on neurodevelopment and 3) identify contextual determinants related to implementing this intervention in a larger cohort. The problem addressed in this proposal is very important. The mentors are outstanding. The environment is excellent and there is documented institutional support for the candidate. However, there are several weaknesses related to methodological issues involving a longitudinal study of neurodevelopment, as well as Aim 3 seems to depend on Aim 2, and there are no alternative strategies provided should Aim 2 be a negative result; these weaknesses dampen overall enthusiasm for this application.

1. Candidate:
Strengths
- Committed to a career in patient oriented research aimed at improving neurodevelopmental outcomes in children with sickle cell disease.
- Excellent research training, completed an OTD in 2010, a PhD in 2019, and a post-doctoral externship in Groningen, The Netherlands in 2019.
- The candidate's PhD work at Washington University was awarded the Tarlov and Ware Doctor Dissertation Award in Patient Reported Outcomes.
- Currently an instructor in the Program of Occupational Therapy at the Washington University School of Medicine.
- Is the site co-PI on a multi-site RCT of intensive infant rehabilitation for children <36 months with perinatal arterial stroke.
- Is a participant in the Mentored Training in Implementation Science program and has 2 internal awards to gather preliminary data.
- Has 12 first author publications.

Weaknesses
- Minimal.

2. Career Development Plan/Career Goals & Objectives:

Strengths

- Has 5 training goals that address gaps in the candidate's knowledge related to implementation science, mixed methods analysis, longitudinal behavioral research design, develop skills in reaching diverse audiences, and develop an independent laboratory.
- Provides a detailed timeline for the training plan that includes deliverables.
- Clearly delineates specific roles for mentor, co-mentors, and advisors in each training goal.

Weaknesses

- Minimal.

3. Research Plan:

Strengths

- Addresses a very important issue in sickle cell disease, neurodevelopmental outcomes and early trajectories of development.
- Will look longitudinally at neurodevelopment at 3 early time-points, 9, 18, and 30 months in a cohort of children with sickle cell disease and in a comparison group.
- Will examine a 12 month home based intervention starting in the first year of life and compare outcomes at 9, 18, and 30 months with those found in the sickle cell group in Aim 1.

Weaknesses

- Given the numbers of infants seen with sickle cell disease, the candidate will need a high rate of consent to complete Aim 1 in 2.5 years. The candidate also states that children who receive therapy services will be analyzed separately, how will this impact recruitment, retention, and the power analyses?
- The longitudinal aspect of assessment is a strength of the application, however in any longitudinal assessment of neurodevelopmental outcomes there is usually a drop-out rate as families move, circumstances change, etc., and the candidate states that there is a 75% adherence to scheduled visits. Yet, there is no discussion of how drop-out will be handled in this study.
- How exactly will the comparison cohort be recruited to be matched based on race, age, sex, socioeconomic status and ADI? Will this cohort be recruited after the sickle cell cohort? Furthermore, how will typically developing be defined in this cohort? There is potential that some of the comparison cohort will have delays discovered at the time of assessment at 9, 18, or 30 months, will these subjects be excluded?
- The candidate states that, "fragile health" is an exclusion criteria, therefore fragile health should be objectively defined. It is likely that more sickle cell subjects will have fragile health and that will likely impact recruitment.
- How will sickle cell subjects that develop stroke after their initial assessment at 9 months of age be handled in the data analysis?
- How will missing data be dealt with in the analyses in Aims 1 and 2?
- Is Aim 3 dependent on Aim 2, that is to say, if the SCCCD intervention has no significant effect on neurodevelopmental assessments, then in this case there probably isn't a need to complete Aim 3. There is no alternative strategy provided for this scenario.
- A minor point, in the study timeline Aim 3 starts in year 2 but Aim 2 (from which the participants in Aim 3 will be recruited) doesn't start until year 3.

4. Mentor(s), Co-Mentor(s), Consultant(s), Collaborator(s):

Strengths

- The candidate identifies a primary mentor, 2 co-mentors and 2 advisors, who have the necessary and complementary expertise to provide outstanding mentorship, and each of the mentors have provided a mentoring plan for the candidate.
- The primary mentor has a long-standing mentoring relationship with the candidate.
- The primary mentor and one of the co-mentors have an impressive record of successful mentoring.
- Dr Yan, a co-mentor on the application, will provide biostatistical mentoring and support.

Weaknesses
- Minimal.

5. Environment and Institutional Commitment to the Candidate:
Strengths
- The environment is excellent.
- The institution is committed to the candidate.

Weaknesses
- Minimal.

Study Timeline:
Strengths
- N/A not a clinical trial.

Weaknesses
- Not Applicable

CRITIQUE 2:

Candidate: 1
Career Development Plan/Career Goals /Plan to Provide Mentoring: 2
Research Plan: 3
Mentor(s), Co-Mentor(s), Consultant(s), Collaborator(s): 1
Environment Commitment to the Candidate: 1

Overall Impact:
This is a resubmission from an occupational therapist with substantial research experience and training. The application has a number of strengths. The candidate has been successful in publication record and now obtaining institutional funding. The mentorship team led by Dr. King is exceptional with respect to research track record and mentoring. The focus on child development among children with SCD (ages 0-3) is highly significant. The research plan is strong. Overall the application is responsive to prior critiques. A couple minor to moderate weaknesses exist in the research plan. The proposal focuses on primary care as the source of developmental screening and referrals. However, children with SCD (assuming access) should have opportunities for screening and referral by hematologists as well. The application does not address the state of screening in hematology care. Lastly, the application doesn't clearly delineate the difference between the candidate's prior pilot intervention study and the study proposed for the K23.

1. Candidate:
Strengths
- The candidate is exceptionally well-trained with PhD in rehabilitation science and postdoc in implementation science.
- The candidate has completed a K12.
- The candidate has a strong publication record with 20 articles.
- The candidate has practical experience as a research coordinator.
- Has received internal awards.
- Received Recognition of Achievement from American Occupational Therapy Association.

Weaknesses
- None

2. Career Development Plan/Career Goals & Objectives:
Strengths

- The candidate's long-term goals are clearly outlined improve screening, diagnosis, and interventions to enhance health, academic, and psychosocial outcomes of children with SCD.
- Training goals appropriate for proposed research - clinical trial design, implementation science, mixed methods and longitudinal data analyses, and skills to lead large-scale multidisciplinary scientific projects.
- Appropriate meeting frequency with mentors.
- Appropriate benchmarks for productivity.

Weaknesses

- Application still does not adequately describe what was gained from prior training in implementation science and current gaps.

3. Research Plan:

Strengths

- The specific aims are well-described.
- The work proposed is hypothesis-driven.
- The candidate presents ample preliminary data informing the current proposal.
- Social determinants are well incorporated into the work proposed.

Weaknesses

- The application focuses on developmental screening only in primary care. However, children with SCD also see hematologists who may initiate referrals as well (e.g., Early Intervention, neuropsychological testing at later ages). The application largely ignores that opportunities for developmental screening exist in two settings for children with SCD which should in theory increase the opportunity for developmental screening.
- The application does not clearly distinguish what is different in this intervention versus the pilot study conducted previously by the candidate which showed feasibility and preliminary effectiveness. Where does this study contribute to the continuum of pilot study and RCT?

4. Mentor(s), Co-Mentor(s), Consultant(s), Collaborator(s):

Strengths

- The primary mentor King is exceptional with a substantial track record in funding, K24, and being a Co-Director of a K12 Mentored Training in Implementation Science Program.
- There is a longstanding mentorship relationship between the candidate and Dr. King.
- The mentorship team demonstrates complementary expertise.

Weaknesses

- None

5. Environment and Institutional Commitment to the Candidate:

Strengths

- Wash U has a very strong research environment and infrastructure.
- The institutional letter is very supportive.

Weaknesses

- None

Study Timeline:

Strengths

- Appropriate

Weaknesses

- None

CRITIQUE 3:

Candidate: 1
Career Development Plan/Career Goals /Plan to Provide Mentoring: 1
Research Plan: 3
Mentor(s), Co-Mentor(s), Consultant(s), Collaborator(s): 1
Environment Commitment to the Candidate: 1

Overall Impact:
This is a resubmission of a K23 application for Dr. Catherin Hoyt, who is an outstanding and unique candidate as an OT with a PhD in rehabilitation science, who has the potential to contribute significantly to the developmental assessment and implementation of early interventions for young children with sickle cell disease. She is a new Instructor of OT at Wash U since July 2021. The research proposed is highly significant in contributing to positive developmental outcomes for children with sickle cell disease. The institutional environment and mentorship team is very strong. Dr. Hoyt has been very responsive to the critiques particularly related to her research plan and approach. She has substantially modified the research design in alignment with the NHLBI vision to improve health and reduce disease. The proposal is substantially improved. Dr. Hoyt shows great potential for development into an independent clinician scientist in a field that is often neglected. Her work may greatly improve quality of life for children with sickle cell disease.

1. Candidate:
Strengths
- PhD in rehabilitation science with interest in SCD.
- Underrepresented group of scientists at NHLBI.
- Participant in Mentored Training in Implementation Science program (NHLBI, K12- HL137942, PI: Davila, King).
- Awarded 2 new internal grants ($100K) since initial submission.
- 22 publications, 11 as first author.
- Description from LOS: "Catherine has a tenacious perseverance to improve the lives of all people with disabilities and she has recognized that research in early identification of deficits may be able to change trajectories of health".

Weaknesses
- None

2. Career Development Plan/Career Goals & Objectives:
Strengths
- K23 goals are well-articulated. The candidate wants to improve screening, diagnosis, and interventions to enhance health, academic, and psychosocial outcomes of children with SCD.
- The candidate clearly identifies training goals: implementation science, qualitative methods, longitudinal study design, publications, leadership.
- Mentored training plan and timeline for completion is excellent with specific benchmarks for each goal.
- # of proposed mentees reduced in order for Dr. Hoyt to focus on her own career development.

Weaknesses
- Not Applicable

3. Research Plan:
Strengths
- High significance: Early developmental delays common in 50% of patients with SCD before age of 3 years.
- Strong preliminary data to support research question.
- Specific Aims modified substantially in response to reviewer comments.

- Improved assessment of social determinants of health (incorporated the area Deprivation Index based on census block etc).
- Inclusion of Patient-Reported Outcomes a Strength (PROMIS) and measure of executive function (BRIEF-P).

Weaknesses

- Concern for feasibility of recruiting 50 children with SCD < 9 months and 50 controls as part of Aim 1. Wash U has a large SCD population and mentorship team can help support the recruitment efforts, however that is an ambitious goal for a rare disease. With 25 newborns annually that translates to ~65 in 2.5 years. The team is unlikely to obtain 75% acceptance for participation.

4. Mentor(s), Co-Mentor(s), Consultant(s), Collaborator(s):
Strengths

- Outstanding mentorship and advisory team; primary mentor (Dr. King) has K24.
- Longstanding collaboration with Dr. King with publication track record.
- Weekly meetings.
- Dr. Yan, an expert in epidemiological statistics, has joined the mentoring team and provided additional feedback and support to adequately address analytic issues (e.g., participant attrition, longitudinal mixed models analyses).

Weaknesses

- None

5. Environment and Institutional Commitment to the Candidate:
Strengths

- Excellent environment to foster career development.
- Funds from primary mentor's K24 supporting early data collection for the candidate; statistical support has been coming from the mentor's K24.
- 75% protected time, strong institutional commitment.

Weaknesses

- None

Study Timeline:
Strengths

- Well planned out, however it may not be feasible to recruit 50 infants with SCD < 9 months of age in 2.5 years.

Weaknesses

- Not Applicable

Resubmission:

- This is a resubmission where the candidate was very responsive to the critiques of the reviewers.

THE FOLLOWING SECTIONS WERE PREPARED BY THE SCIENTIFIC REVIEW OFFICER TO SUMMARIZE THE OUTCOME OF DISCUSSIONS OF THE REVIEW COMMITTEE, OR REVIEWERS' WRITTEN CRITIQUES, ON THE FOLLOWING ISSUES:

PROTECTION OF HUMAN SUBJECTS (RESUME): ACCEPTABLE

INCLUSION OF WOMEN PLAN (RESUME): ACCEPTABLE

INCLUSION OF MINORITIES PLAN (RESUME): ACCEPTABLE

INCLUSION OF AGE ACROSS THE LIFESPAN (RESUME): ACCEPTABLE

TRAINING IN THE RESPONSIBLE CONDUCT OF RESEARCH: ACCEPTABLE

RESOURCE SHARING PLANS: ACCEPTABLE

AUTHENTICATION OF KEY BIOLOGICAL AND/OR CHEMICAL RESOURCES: Not applicable

COMMITTEE BUDGET RECOMMENDATIONS: RECOMMENDED AS REQUESTED

Footnotes for 1 K23 HL161328-01A1; PI Name: Hoyt, Catherine Rose

NIH has modified its policy regarding the receipt of resubmissions (amended applications).See Guide Notice NOT-OD-18-197 at https://grants.nih.gov/grants/guide/notice-files/NOT-OD-18-197.html. The impact/priority score is calculated after discussion of an application by averaging the overall scores (1-9) given by all voting reviewers on the committee and multiplying by 10. The criterion scores are submitted prior to the meeting by the individual reviewers assigned to an application, and are not discussed specifically at the review meeting or calculated into the overall impact score. Some applications also receive a percentile ranking. For details on the review process, see http://grants.nih.gov/grants/peer_review_process.htm#scoring.

MEETING ROSTER

NHLBI Mentored Patient-Oriented Research Study Section
Heart, Lung, and Blood Initial Review Group
NATIONAL HEART, LUNG, AND BLOOD INSTITUTE
MPOR (OA)
06/30/2022 - 07/01/2022

Notice of NIH Policy to All Applicants: Meeting rosters are provided for information purposes only. Applicant investigators and institutional officials must not communicate directly with study section members about an application before or after the review. Failure to observe this policy will create a serious breach of integrity in the peer review process, and may lead to actions outlined in NOT-OD-22-044 at https://grants.nih.gov/grants/guide/notice-files/NOT-OD-22-044.html, including removal of the application from immediate review.

CHAIRPERSON(S)

ANDERSON, CHERYL A. M., PHD
DEAN
HERBERT WERTHAM SCHOOL OF PUBLIC HEALTH &
HUMAN LONGEVITY SCIENCE
SCHOOL OF MEDICINE
UNIVERSITY OF CALIFORNIA, SAN DIEGO
LA JOLLA, CA 92093

MEMBERS

ALLEN, AMANI M., PHD
ASSOCIATE PROFESSOR
DEPARTMENT OF COMMUNITY HEALTH SCIENCES
DIVISION OF EPIDEMIOLOGY
UNIVERSITY OF CALIFORNIA, BERKELEY
BERKLEY, CA 94720

BENZO, ROBERTO P., MD
ASSOCIATE PROFESSOR
DEPARTMENT OF INTERNAL MEDICINE
DIVISION OF PULMONARY & CRITICAL CARE
MAYO CLINIC
ROCHESTER, MN 55902

CHRISTIE, JASON D., MD
PROFESSOR
DEPARTMENT OF MEDICINE
DIVISION OF PULMONARY AND CRITICAL CARE MEDICINE
CENTER FOR CLINICAL EPIDEMIOLOGY AND BIOSTATISTICS
UNIVERSITY OF PENNSYLVANIA
PHILADELPHIA, PA 19104

DAMARLA, MAHENDRA, MD
ASSOCIATE PROFESSOR
DEPARTMENT OF PULMONARY DISEASES
DIVISION OF CRITICAL CARE MEDICINE
JOHNS HOPKINS UNIVERSITY
BALTIMORE, MD 21224

DAVIS, STEPHANIE D., MD
CURNEN PROFESSOR/CHAIR
DEPARTMENT OF PEDIATRICS
UNC HEALTH CHILDREN'S
UNIVERSITY OF NORTH CAROLINA AT CHAPEL HILL
CHAPEL HILL, NC 27599

EVERHART, ROBIN S., PHD
ASSOCIATE PROFESSOR
DEPARTMENT OF HEALTH PSYCHOLOGY
VIRGINIA COMMONWEALTH UNIVERSITY
RICHMOND, VA 23284

FLOOD, VERONICA H., MD
PROFESSOR
DEPARTMENT OF HEMATOLOGY/ONCOLOGY
DIVISION OF BLOOD RESEARCH INSTITUTE
MEDICAL COLLEGE OF WISCONSIN
MILWAUKEE, WI 53226

FOLTA, SARA, PHD *
ASSOCIATE PROFESSOR
FRIEDMAN SCHOOL OF NUTRITION
SCIENCE AND POLICY
TUFTS UNVIERSITY
BOSTON, MA 02111

GLASSBERG, JEFFREY A., MD
ASSOCIATE PROFESSOR
EMERGENCY MEDICINE
HEMATOLOGY & MEDICAL ONCOLOGY
MOUNT SINAI COMPREHENSIVE SICKLE CELL PROGRAM
ICAHN SCHOOL OF MEDICINE AT MOUNT SINAI
NEW YORK, NY 10029

HILEMAN, CORRILYNN O., MD *
CLINIC MEDICAL DIRECTOR
DEPARTMENT OF INTERNAL MEDICINE
DIVISION OF INFECTIOUS GENERAL DISEASE
METROHEALTH MEDICAL CENTER
CLEVELAND, OH 44109

HO, JENNIFER EN-SIAN, MD *
ASSOCIATE PROFESSOR
DIVISION OF CARDIOVASCULAR MEDICINE
HEART FAILURE & TRANSPLANTATION
BETH ISRAEL DEACONESS MEDICAL CENTER
BOSTON, MA 02114

HOFFMAN, LUCAS R., MD, PHD
PROFESSOR
DEPARTMENT OF PEDIATRICS
DIVISION OF PULMONARY AND SLEEP MEDICINE
SEATTLE CHILDREN'S HOSPITAL
UNIVERSITY OF WASHINGTON
SEATTLE, WA 98105

HOUGH, CATHERINE L., MD *
PROFESSOR
DEPARTMENT OF PULMONARY
& CRITICAL CARE MEDICINE
OREGON HEALTH & SCIENCE UNIVERSITY
PORTLAND, OR 97239

LEWIS, CHRYSTAL L., PHD *
ASSOCIATE PROFESSOR
USA COLLEGE OF NURSING
ADULT HEALTH NURSING
UNIVERSITY OF SOUTH ALABAMA
MOBILE, AL 36693

LINDER, MARK W., PHD
PROFESSOR
DEPARTMENT OF PATHOLOGY
AND LABORATORY MEDICINE
UNIVERSITY OF LOUISVILLE
LOUISVILLE, KY 40299

MIYAMOTO, SHELLEY D., MD
ASSOCIATE PROFESSOR
DEPARTMENT OF PEDIATRICS-CARDIOLOGY
CHILDREN’S HOSPITAL COLORADO
UNIVERSITY OF COLORADO
AURORA, CO 80045

MORRIS, CLAUDIA R., MD
PROFESSOR
DEPARTMENT OF PEDIATRICS
DIVISION OF EMERGENCY MEDICINE
CENTER FOR CLINICAL AND TRANSLATIONAL RESEARCH
EMORY UNIVERSITY
ATLANTA, GA 30322

MORRIS, PETER E., MD *
PROFESSOR
DEPARTMENT OF INTERNAL MEDICINE
DIVISION OF PULMONARY, ALLERGY & CRITICAL CARE
UNIVERSITY OF ALABAMA AT BIRMINGHAM
BIRMINGHAM, AL 35294

NELIN, LEIF D., MD
DIVISION CHIEF
CENTER FOR PERINATAL RESEARCH
NATIONWIDE CHILDREN'S HOSPITAL
OHIO STATE UNIVERSITY
COLUMBUS, OH 43215

OLDHAM, JUSTIN M., MD *
ASSOCIATE PROFESSOR
DEPARTMENT OF PULMONARY
DIVISION OF CRITICAL CARE MEDICINE
UNIVERSITY OF MICHIGAN
ANN ARBOR, MI 48103

PIPINOS, IRAKLIS ILIAS, MD, PHD
PROFESSOR
DEPARTMENT OF SURGERY
UNIVERSITY OF NEBRASKA MEDICAL CENTER
OMAHA, NE 68198

RAPHAEL, JEAN L., MD *
DIVISION CHIEF/PROFESSOR
DEPARTMENT OF PEDIATRICS ACADEMIC GENERAL
TEXAS CHILDREN'S HOSPITAL, CLINICAL CARE CENTER
CHILD HEALTH POLICY & ADOVACY
BAYLOR COLLEGE OF MEDICINE
HOUSTON, TX 77030

REYES-SOFFER, GISSETTE, MD
ASSISTANT PROFESSOR
DEPARTMENT OF MEDICINE
COLLEGE OF PHYSICIANS & SURGEONS
COLUMBIA UNIVERSITY IRVING MEDICAL CENTER
NEW YORK, NY 10032

SAN JOSE ESTEPAR, RAUL, PHD
ASSOCIATE PROFESSOR
DEPARTMENT OF RADIOLOGY
BRIGHAM AND WOMEN'S HOSPITAL
HARVARD MEDICAL SCHOOL
BOSTON, MA 02115

SENGUPTA, PARTHO P., MD
CHIEF
DEPARTMENT OF CARDIOLOGY
RUTGERS ROBERT WOOD JOHNSON MEDICAL SCHOOL
RUTGERS UNIVERSITY
NEW BRUNSWICK, NJ 08901

SHENOY, CHETAN N., MS *
ASSOCIATE PROFESSOR
DEPARTMENT OF INTERNAL MEDICINE
DIVISION OF CARDIOVASCULAR
UNIVERSITY OF MINNESOTA
MINNEAPOLIS, MN 55455

SHOEMAKER, MOORE B., MD *
ASSOCIATE PROFESSOR
DEPARTMENT OF CARDIOLOGY
ADULT CONGENITAL HEART DISEASE
CARDIAC ELECTROPHYSIOLOGY
VANDERBILT UNIVERSITY MEDICAL CENTER
NASHVILLE, TN 37212

SINGER, JONATHAN P., MD *
DIRECTOR/ASSOCIATE PROFESSOR
DIVISION OF PULMONARY
& CRITICAL SLEEP MEDICINE
UNIVERSITY OF CALIFORNIA, SAN FRANCISCO
SAN FRANCISCO, CA 94143

SINGLA, DINENDER KUMAR, PHD
PROFESSOR/HEAD
DIVISION OF METABOLIC & CARDIOVASCULAR SCIENCES
BURNETT SCHOOL OF BIOMEDICAL SCIENCES
UNIVERSITY OF CENTRAL FLORIDA
ORLANDO, FL 32816

SNYDER, EDWARD L., MD *
PROFESSOR/DIRECTOR
DEPARTMENT OF LABORATORY MEDICINE
& BLOOD BANK
YALE-NEW HAVEN HOSPITAL
YALE UNIVERSITY
NEW HAVEN, CT 06504

STONE, KATIE L., PHD *
SENIOR SCIENTIST
SAN FRANCISCO COORDINATING CENTER
RESEARCH INSTITUTE
CALIFORNIA PACIFIC MEDICAL CENTER
SAN FRANCISCO, CA 94107

TUTTLE, KATHERINE R., MD
EXECUTIVE DIRECTOR
PROVIDENCE MEDICAL RESEARCH CENTER
PROVIDENCE HEALTH CARE
UNIVERSITY OF WASHINGTON
SPOKANE, WA 98195

WANG, THOMAS J., MD *
PROFESSOR/CHAIR
DEPARTMENT OF INTERNAL MEDICINE
DIVISION OF CARDIOLOGY
UT SOUTHWESTERN MEDICAL CENTER
5323 HARRY HINES BOULEVARD
DALLAS, TX 75390

WILLIAMS, JONI STROM, MD *
DEPARTMENT OF MEDICINE
DIVISION OF GENERAL INTERNAL MEDICINE
MEDICAL COLLEGE OF WISCONSIN
10361 WEST INNOVATION DRIVE, SUITE 2100
MILWAUKEE, WI 53226

SCIENTIFIC REVIEW OFFICER

WEBB, STEPHANIE JOHNSON, PHD
SCIENTIFIC REVIEW OFFICER
OFFICE OF SCIENTIFIC REVIEW/DERA
NATIONAL HEART, LUNG, AND BLOOD INSTITUTE
NATIONAL INSTITUTES OF HEALTH
BETHESDA, MD 20892

EXTRAMURAL SUPPORT ASSISTANT

MCCORKLE, ATHENA M., BA
EXTRAMURAL SUPPORT ASSISTANT
OFFICE OF SCIENTIFIC REVIEW/DERA
NATIONAL HEART, LUNG, AND BLOOD INSTITUTE
NATIONAL INSTITUTE OF HEALTH
BETHESDA, MD 20892

OBSERVER

COPE, KEARY A, PHD
SCIENTIFIC REVIEW OFFICER
OFFICE OF SCIENTIFIC REVIEW/DERA
NATIONAL HEART, LUNG, AND BLOOD INSTITUTE
BETHESDA, MD 20892

OTHER REVIEW STAFF

NG, THEO E.
NIH PATHWAY INTERN
OFFICE OF SCIENTIFIC REVIEW/DEA
NATIONAL HEART, LUNG, AND BLOOD INSTITUTE
NATIONAL INSTITUTES OF HEALTH
BETHESDA, MD 20892

SMITH, DONYA'E M.
NIH PATHWAYS INTERN
OFFICE OF SCIENTIFIC REVIEW/DERA
NATIONAL HEART, LUNG, AND BLOOD INSTITUTE
NATIONAL INSTITUTES OF HEALTH
BETHESDA, MD 20892

* Temporary Member. For grant applications, temporary members may participate in the entire meeting or may review only selected applications as needed.

Consultants are required to absent themselves from the room during the review of any application if their presence would constitute or appear to constitute a conflict of interest.
